# Single Figure Publications: Towards a novel alternative format for scholarly communication

**DOI:** 10.12688/f1000research.6742.1

**Published:** 2015-07-17

**Authors:** Long Do, William Mobley

**Affiliations:** 1 Department of Neurosciences, University of California, San Diego, CA, 92093, USA

**Keywords:** Single figure paper, Nano-publications, Communication, Publishing

## Abstract

The single figure publication is a novel, efficient format by which to communicate scholarly advances. It will serve as a forerunner of the nano-publication, a modular unit of information critical for machine-driven data aggregation and knowledge integration.

## Introduction

Information is the principal product of research. It comes in a variety of types, as defined by the research discipline and tools used. Information can be used in many different ways - from informing fundamental work to translating basic advances into applications. There is growing sentiment in the scientific community that the current paradigm of scholarly communication is outdated and inefficient ^1– 4^. While the traditional journal article will continue - at least in the near term - to represent the primary public record of new research results, its format in some ways hinders the dissemination of knowledge. First, in representing the outcome of studies done over years of work, the journal article fails to rapidly communicate the results of the various pieces of the larger study, in so doing delaying an opportunity for the research community to benefit from novel findings from individual experiments that may impact ongoing studies. Second, tailored for human comprehension, the journal article is designed to aggregate units of knowledge in a format that is not easily machine-readable; in so doing, it fails to allow for ready integration of information within or across disciplines. Thus, the journal article, while serving a valuable purpose, is not the only means by which to communicate research advances, nor does it optimally serve the research community as currently structured.

## Revitalising scholarly communication; what is required?

An optimal format for scholarly communication would ensure that the information is: 1) valid, as assessed using one or more processes to ensure that what is being communicated has been objectively vetted; 2) complete, such that all of the methods and, if applicable, reagents are defined and made available; 3) rapidly shared, admitting of no delays not inherent in the technology used to share; 4) easily challenged, by those whose research findings are in disagreement; 5) readily interrogated using novel approaches to informatics that allows for integration across disciplines; 6) free of charge, or as free as possible; and 7) free of bias. The latter deserves amplification. Not only should the researcher be free of bias with respect to the findings communicated, so should the reviewer(s) who decides upon its publication. Indeed, it is important that publication of findings should to the greatest extent possible also be free of bias on the basis of reviewer estimates of ‘impact’. We may think we know what is important and what is not, and our experience may back up our view, but only the test of time can show which research findings were the most influential. Inhibiting the exchange of information of perceived ‘low impact’ is as objectionable as excessively emphasizing the publication of information of perceived ‘high impact’. Giving the research community all of the data in a form that can be readily communicated and validated empowers each of its participants to decide what findings are impactful.

## Nano-publications: a step in the right direction

It is our view that emerging technologies can be used to enhance the effectiveness of scholarly communication. To increase the speed and efficiency of knowledge dissemination, the computational and informatics community has proposed the use of nano-publications - modular, nimble units of communication designed to be machine-readable. Nano-publications as currently defined by the World Wide Web Consortium (W3C), are validated, core scientific statements, with associated context. However, implementation and adoption of the nano-publication has thus far faced many challenges. Common strategies to generate nano-publications have focused on extracting them from the existing literature in a “top-down” fashion, by means of either manual curation or automated text mining. Neither method is sustainable, thus limiting the use of this top down approach. Using existing tools for text mining the large collection of data in a traditional journal article results in a data analysis burden that is unmanageable. Moreover, existing methods directed at the traditional article can result in predictions that are difficult to properly curate and validate.

## The Single Figure Publication

We offer the idea of the micro-publication unit, the single figure publication (SFP), to provide scholars with a real-world, manageable method to inform research. The SFP, consisting of a figure, the legend, the Material and Methods section, and an optional Results/Discussion section, reduces the unit of publication to a more tractable size. Importantly, it results in a markedly decreased time from data generation to publication. As such, SFPs represent a new means by which to communicate scientific research. As with the traditional journal article, the content of the SFPs is readily understandable by the scientist. Coupled with additional tools that aid in structuring content (e.g. describing in detail the methods using pre-defined steps from protocols), the SFP represents a “bottom-up” means by which scholars can structure the content of their findings in a modular and piece-wise fashion wedded to everyday laboratory life.

A significant additional benefit is that the SFP represents an important bridge between the traditional journal article and the nano-publication. Nano-publications principally serve the need to integrate and aggregate data. Because the research community will increasingly desire to examine data in large volumes of a variety of types, nano-publications will become essential. However, nano-publications do not represent an optimal medium for human communication and consumption. These publications are structured to be read by machines, not people. For these reasons, nano-publications alone cannot serve to inform the scholarly community.

## Challenges faced

Successfully implementing a new publication infrastructure – i.e. one that extends from the traditional publication and the SFP to the nano-publication - will require the development of tools and a coherent strategy to promote social change in the scientific community. Changing the social ecosystem of scholarly communication to implement the benefits of information technology has met hurdles and resistance. Scholars are wedded to the existing formats and the reward systems in academia depend on those delivery mechanisms. Investigators desire maximum visibility that is currently secured by publishing in journals with high impact factors. This practice is unlikely to change because our colleagues (and as a result, our institutions and granting agencies) judge the importance of work by the journals in which it is published. An investigator thus ignores the quest for publishing in such journals at her/his peril with respect to grant funding and promotion. At best our behavior respects the fact that we cannot read every paper published by our colleagues and thus rely on the expertise of peer reviewers and editors who understand research and deserve our respect and confidence. At worst, we delegate to others the responsibility of judging the creativity of our colleagues and the importance of their work.

The SFP inherently provides for the complete, unbiased and rapid publication in a forum that encourages active questioning and subsequent validation and at a cost that is a tiny fraction of many other modes of publication. While the SFP thus addresses each of the objectives outlined above for exchanging information, it is unlikely to become a dominant mode for information exchange, at least in the near term. A number of factors contribute; at present, investigators publish the results of many experiments focused on one or more questions. The net result is a whole ‘story’ that provides a complete look at a topic. A collection of SFPs published in series and then pulled together in a comprehensive package, or album, that provides a more thorough analysis across individual experiments would accomplish the same end. But it is an open question as to whether or not publishers or researchers would choose this path. As one option, it would be possible for publishers to encourage authors whose full length journal articles are chosen for publication to also submit individual SFPs that make up the article. This would support the eventual creation of nano-publications of the work, providing benefit to the publisher, the authors and the community.

## Finding a niche: the future of Single Figure Publications

Given the challenges to the SFP, where might it most readily make a contribution at this time? We envision a number of benefits. First, it can be a valuable medium for communicating information that is unlikely to find its way into a traditional publication. Most laboratories have conducted studies that, while interesting and worthy of communication, are incomplete and not likely to be accepted for publication. Rather than spending the time and resources needed to bring the work to completion, the SFP provides an excellent platform to communicate valid findings. A second niche for the SFP is as a medium for informing investigators about methods and reagents. As an example, those of us who purchase antibodies have spent precious resources on reagents that do not perform as indicated by the manufacturer. An SFP that publishes a laboratory’s experience would save money and effort. Ideally, it would encourage manufacturers to more carefully validate the performance of the reagents they sell. A third niche is very important, but nearly impossible to publish: negative studies. Just because data are published, even prominently, this does not mean that they are reproducible. The research community understands this but has relatively few options for addressing the issue effectively. Word of mouth is a poor substitute for clear exposition. Here again, the SFP could save time and energy that will otherwise be spent in trying unsuccessfully to validate findings.

We view the SFP as a valuable and necessary part of the infrastructure required to achieve the goals of scholarly communication. While the traditional format of journal articles will continue to be used to tell the important ‘stories’ of scientific journeys, more nimble, modular units of communication are needed, starting with SFPs. The emergence of additional tools that help structure content while authoring SFPs will facilitate the creation of nano-publications, thus allowing us to put machines to work in the service of informing and enhancing our science.

Accordingly, we encourage publishers to offer the SFP as an option for publication and look forward to the emergence of the SFP as increasing efforts to develop the tools required for researchers to turn ideas and results into structured concepts. Indeed, in part owing to the use of the SFP format, we imagine a future environment for publishing that is considerably more dynamic and delivers information and resulting insights far more effectively.
